# Understanding Inequalities of Maternal Smoking—Bridging the Gap with Adapted Intervention Strategies

**DOI:** 10.3390/ijerph13030282

**Published:** 2016-03-04

**Authors:** Julie Boucher, Anne T. M. Konkle

**Affiliations:** 1 Interdisciplinary School of Health Sciences, University of Ottawa, Ontario, ON K1N 6N5, Canada; Anne.Konkle@uOttawa.ca; 2 School of Psychology, University of Ottawa, Ontario, ON K1N 6N5, Canada

**Keywords:** smoking cessation, pregnancy, socioeconomic status, dependence, social support, culture, mental health, health services

## Abstract

Women who are generally part of socially disadvantaged and economically marginalized groups are especially susceptible to smoking during pregnancy but smoking rates are underreported in both research and interventions. While there is evidence to support the short-term efficacy of nicotine replacement therapy (NRT) use in pregnancy, long-term abstinence rates are modest. Current health strategies and interventions designed to diminish smoking in pregnancy have adopted a simplified approach to maternal smoking—one that suggests that they have a similar degree of choice to non-pregnant smokers regarding the avoidance of risk factors, and overlooks individual predictors of non-adherence. As a result, interventions have been ineffective among this high-risk group. For this reason, this paper addresses the multiple and interacting determinants that must be considered when developing and implementing effective strategies that lead to successful smoking cessation: socioeconomic status (SES), nicotine dependence, social support, culture, mental health, and health services. Based on our review of the literature, we conclude that tailoring cessation programs for pregnant smokers may ultimately optimize NRT efficacy and reduce the prevalence of maternal smoking.

## 1. Introduction

Although the proportion of women who smoke during pregnancy in high-income countries has declined, it remains an international public health priority. The economic burden of tobacco-related morbidity and mortality is substantial [1,2], contributing significantly to socioeconomic inequalities in stillbirths and infant deaths (38% and 31% respectively), as shown in a retrospective cohort study of mothers with varying degrees of socioeconomic deprivation [3]. This is in accordance with an observational study by Bauld, Judge and Platt [4], whereby despite low overall efficacy, smoking cessation services have had a disproportionate effect in the most disadvantaged groups, possibly reducing the social gradient. Unfortunately, there is little information available regarding smoking cessation treatment efficacy in pregnancy and the few relevant studies provide conflicting results [5].

According to the Smoking and Nicotine in Pregnancy (SNAP) trial, the largest nicotine replacement therapy (NRT) randomized control trial conducted in pregnancy thus far, smoking cessation treatment failure in pregnancy may stem from a low adherence rate [6]. Of the 981 participants followed up at delivery, only 7.2% (35/485) of women assigned to receive NRT and 2.8% (14/496) assigned to receive placebo, reported using trial medications for more than one month. This outcome has been corroborated in numerous smaller studies, where mean duration of NRT use rarely exceeded one month [5,7,8]. 

Experts agree that the increased nicotine clearance from the body experienced during pregnancy is partly to blame for lack of compliance to an NRT regime and higher doses of nicotine may be required to optimize efficacy [9]. 

Nevertheless, promising results were reported in a randomized trial in which a combination of cognitive behavioural therapy (CBT) and NRT increased cessation rates nearly threefold compared to CBT alone [8]. However, recruitment was stopped early due to higher risk of negative birth outcomes in the CBT + NRT group, which was later reported to have resulted from a greater history of preterm births in the CBT + NRT group.

In spite of this, many research trials and treatment interventions disregard the complex reasons for women’s smoking patterns that warrant the use of CBT and tailored interventions. Barriers to quitting are multiple and interacting psychosocial, cultural, economic, and biological influences, further accentuated in pregnancy and postpartum, that factor into the behaviour that goes beyond physiological dependence. Unlike persistent smokers, spontaneous quitters tend to be more highly educated, less addicted, and less likely to have partners who smoke [10]. Yet, interventions tend to focus on individual behaviour change [11], overlooking important predictors of non-adherence such as the level of education, baseline cotinine levels [12], psychosocial barriers [13], culture [14], and more. 

The purpose of the present article is to explore overall patterns of results across studies of maternal smoking and smoking cessation, thus isolating the determinants of health that affect the likelihood of quitting during pregnancy. This is not a systematic literature review, but rather a summary of peer-reviewed publications highlighting the disparities of maternal smoking in Canada and other high-income countries. We focus on six key factors that are predictive of maternal smoking: socioeconomic status (SES), nicotine dependence, social support, culture, mental health, and health services. 

## 2. Determinants of Health

### 2.1. Socioeconomic Status 

High-income countries have seen a significant decline in maternal smoking rates since the 1980s [15,16]. Unfortunately, this is not uniform across all sectors of society. Low socioeconomic groups have experienced a much slower rate of decline relative to those of higher socioeconomic standing [16]. In Canada, smoking prevalence is higher among pregnant women with low household income, who are less than university educated, and who have not recently held a job [17]. While all three of these variables are not always represented, there is a clear pattern arising from Canadian studies [18,19,20]. Regardless of how social status is operationalized (low income, low educational attainment, low occupational status), these findings are also consistent with those documented in other developed countries including Australia [21,22], Iceland [23], USA [24,25,26], Scotland [27], and Finland [28].

Cigarette smoking is a marker of social disadvantage in high-income countries and has been cited as one of the most important contributing factors of health inequality between the rich and poor [29]. Lower-SES women tend to have more psychological and emotional problems, less social support, fewer financial resources, and less residential stability [30,31]. Pregnancy also brings about added stress and smoking is a perceived stress reducer. The World Health Organization’s report *Social Determinants of Health* acknowledges that disadvantaged people are more likely to use substances in response to their circumstances [32] and yet, up until recently, smoking-cessation strategies and interventions failed to address the social barriers to participation among these high-risk groups. For example, a Canadian study conducted by Stewart and colleagues [33] found that only 23% of treatment programs geared towards women were appropriate for, or accessible to, disadvantaged smokers. Higher cigarette consumption, lower educational level, higher confidence in ability to quit on one’s own, and multiparity, are some of the risk factors that have predicted treatment non-adherence among pregnant women with low SES [13].

This overrepresentation of women with lower SES among pregnant smokers warrants the need for approaches that address modifiable risk factors of non-adherence. It is imperative that health professionals acknowledge the difficulties encountered by underserved minority pregnant smokers when developing and implementing cessation strategies such as lack of childcare, transportation, psychosocial barriers [13], insufficient knowledge of health risks and cessation methods, and lack of culturally appropriate quit support [14]. In order to so, anti-smoking interventions will need to adopt a positive rather than punitive approach and respect individual values, capabilities and circumstances to achieve compliance in women [13,14]. Short-term strategies may involve, for example, providing childcare or utilizing telecommunication technologies for intervention delivery, to ensure that women have access to proper prenatal, perinatal and postnatal care [13]. Healthcare professionals should also be tasked with increasing knowledge of smoking harms and cessation methods, as well as providing culturally competent care to low-SES groups. Long-term strategies should aim to de-normalize smoking, examine causes of resistance to antitobacco messages, and investigate the roles of stress and depression in maternal smoking behaviour [14]. 

### 2.2. Nicotine Dependence

A retrospective study conducted in British-Columbia reported that smoking during pregnancy is a significant dose-dependent risk factor for adverse birth outcomes including small-for-gestational age, low birth weight at term, and intra-uterine growth restriction [19]. 

There appears to be a consensus whereby maternal smoking is more prevalent among women smoking heavily prior to pregnancy, as measured by nicotine dependence scales as well as the number of cigarettes smoked in a day [17,21,34]. Johnson *et al*. [20] found that Canadian women smoked on average 9.6 cigarettes daily during the first trimester, exceeding the 7.5 cigarettes smoked by women who quit when they found out they were pregnant. This would suggest that spontaneous quitters have a lower degree of nicotine dependence and therefore have less difficulty quitting than heavy smokers [22,35]. However, the reported number of cigarettes smoked across pregnancy, was lower than the average daily consumption reported by non-pregnant female smokers in Ontario (15.2 cigarettes) [36]. These data could be skewed due to bias—whether recall or underreporting due to social stigmatization—or it could be an accurate representation of smoking status among women who, although unable to quit, reduce tobacco consumption as a precaution while pregnant [20]. Data from the SNAP trial demonstrated that this association persisted throughout pregnancy [12]. Women with lower baseline cotinine levels were more likely to achieve cessation 1 month after the quit date and at delivery, than women with a higher baseline cotinine. 

This relationship between heavy smoking and continued smoking during pregnancy has also been reported across developed countries, in Australia [21,22], Canada [17], USA [26], and France [37].

A review of NRT use in pregnancy suggests that, although behaviour modification therapy should be the first course of action when addressing maternal smoking, women with moderate to high levels of dependence who are unable to quit otherwise, should be offered NRT [38]. An observational study by Brose *et al*. [39] went even further, suggesting that combination NRT is more effective than single form NRT at helping women quit smoking during pregnancy. Thus, higher levels of nicotine and faster acting NRT products may be needed to attain an effect that would be sufficient to help in smoking cessation. Although it bears mentioning that outcomes were not measured past the 4-week follow-up; therefore long-term efficacy could not be assessed. 

A recent systematic review also showed promising results, with NRT use increasing cessation rates by approximately 40% in late pregnancy [40]. However, Coleman *et al*. remarked that non-placebo RCTs had a higher risk of bias than placebo-controlled trials; therefore efficacy findings should be interpreted with caution. 

Nevertheless, the evidence is conflicting. The Berlin group [41], who adjusted NRT doses to saliva cotinine levels to match those obtained when smoking, found that NRT did not increase cessation rates compared to placebo in pregnant women. This may be explained in part by the fact that researchers failed to account for the increased nicotine metabolism experienced in pregnancy [42]. Although doses were adjusted 2 weeks after the scheduled quit date, this delay may have led to NRT underdosing. 

It is possible that NRT efficacy varies based on individual differences such as the level of addiction. Two reviews of NRT during pregnancy agree that pregnant women smoking fewer than five cigarettes *per* day would benefit more from behavioural support than NRT, while moderate and heavy smokers should use NRT [38,43].

While standard NRT use in pregnancy does not lead to either positive or negative birth outcomes compared to controls, researchers suggest future RCTs investigate the safety of higher NRT doses [40]. Nevertheless, nicotine coupled with the thousands of chemicals present in cigarette smoke is likely far more harmful than NRT. When used properly, the level of nicotine to which the fetus is exposed is lower than that of cigarettes [44,45]. The Society of Obstetricians and Gynecologists of Canada advises using the lowest effective dose of NRT such as in gum or nasal spray, which provide an intermittent dose of nicotine instead of the transdermal patch, which offers a continuous dose [46]. On the chance that the transdermal patch is used, guidelines advise women to remove it at night to avoid any unnecessary nicotine exposure. Lastly, NRT should be discontinued if the patient resumes smoking at the rate of pre-NRT use. Ultimately, complete avoidance of all nicotine products is the objective in pregnancy and while breastfeeding. But in cases where women fail to quit, NRT use is justifiable in relation to the risk of continued smoking.

In addition, studies suggest that mothers with heavier cigarette consumption are more likely to be included in the following categories: Aboriginal status, low SES [22], low level of education, single parent, drug or alcohol use [19], poor antenatal care attendance, and multiparity [19,22], further supporting the notion of multiple and interacting barriers to smoking cessation.

### 2.3. Social Support

Numerous studies have highlighted the importance of social support networks in helping pregnant women reduce or cease smoking. The likelihood of maternal smoking is associated with the closeness of the tie between parents at the time of the birth [47]. Marital status (an indicator of social support) is highly predictive of successful smoking cessation during pregnancy. Thus, the extent of maternal smoking is lowest in married mothers; followed by cohabiting mothers, then solo mothers. Among non-married women, the closeness of the tie between parents can predict smoking in pregnancy. Particularly noteworthy is the trend whereby an increasing risk of maternal smoking is associated with decreasing parental bonding. Solo mothers not in a relationship with the father have a higher risk of continued smoking compared to those closely involved with the father at the time of the birth. While it may be a relatively new area of research, a recently published article revealed that same-sex cohabiters, like their different-sex cohabiting counterparts, also report a higher risk of smoking prevalence and frequency compared to different-sex married couples [48].

Al-Sahab and colleagues [18] have corroborated the finding that single mothers are at an increased risk of smoking during pregnancy. The Millennium Cohort Study conducted among 18,225 women in the UK also came to this conclusion, revealing that married women have much lower rates of smoking throughout pregnancy compared to cohabitating and single mothers [49]. The presence of a partner reduces the amount smoked by 36% compared to women who did not report any partner involvement [50]. 

In addition to the presence of a partner, the amount of support a woman receives from her significant other is predictive of her likeliness to quit smoking. A supportive husband or stable partner will increase the chances of successful smoking cessation for pregnant women [51,52,53,54]. Accordingly, there is evidence that pregnant smokers are more likely to have challenging relationships and that this pattern of behaviour may interfere with the effectiveness of current public health cessation interventions [55]. 

Pregnant women subjected to physical abuse report a higher use of tobacco, alcohol, and illicit drugs [56,57], suggesting that heightened conflict in a relationship may intensify the urge to smoke and hinder cessation attempts. Challenging interpersonal relationships within the family of origin, peers and neighbours are also more prevalent among smokers compared to quitters and non-smokers [49]; a single problem within interpersonal relationships increases the likelihood of a pregnant woman becoming a persistent heavy smoker by 67%.

Even among women that experience positive social relationships, living with others who smoke is one of the primary barriers to smoking cessation in pregnancy [58,59] because the other smokers provide easy access to cigarettes [60]. Approximately 80% of pregnant smokers are partnered with an expectant father who continues to smoke [61], 78% have a smoker in the household, and 95% have a smoker among friends [21]. Similarly, a cohabitating smoking partner is a risk factor for postpartum relapse [53]. 

In view of the high smoking rates among other household members and friends, smoking cessation interventions must include individuals closely associated with the expectant mothers, particularly partners. Careful consideration must also be given to the power dynamics that exist within these relationships. In the instance that cessation during pregnancy leads to increased partner conflict, interventions must ensure that women are not influenced by their partners, but rather, exert control over their own tobacco reduction experience [62]. A “delinked yet couple-focused” approach has also been suggested, whereby women and their partners receive care separately and work towards creating a supportive environment for smoking cessation [63]. 

For the most part, existing interventions disregard the utility of partner inclusion during research and intervention development. A systematic review of interventions to enhance partner support as part of stop-smoking therapies revealed a lack of effective programs that include or target partners. Nevertheless, the authors have highlighted promising evidence for future intervention avenues that necessitate further investigation (multicomponent interventions, intensity of intervention, delivery of intervention by someone other than pregnant spouse, and tailoring of program for specific population needs) [64]. 

When developing a partner inclusive program, it is also important to tailor them to gender-specific perceptions of smoking and smoking cessation. Qualitative studies have shown that men view smoking as an expression of their masculinity, independence and risk-taking [63]. Consequently, they are reluctant to utilize cessation resources and unable to view their partner’s tobacco cessation attempts for pregnancy as an opportunity to quit themselves. With this in mind, cessation interventions should appeal to expectant fathers by taking into account these social constructs of smoking behaviour. During the transition to fatherhood, men feel especially uncomfortable with their smoking behaviour, which provides healthcare workers an opportunity to target them for cessation interventions.

Overall, quit smoking programs do not address the complex psychosocial context of tobacco use and rely on women having the social resources to independently implement and sustain the necessary behavioural strategies that lead to quitting [49]. They fail to consider that persistent smokers generally have fewer social resources on which to draw and those available to them might be inadequate or themselves be a source of stress and conflict.

### 2.4. Culture

High maternal smoking rates among Indigenous minority groups are largely patterned by their social and material deprivation. Numerous qualitative studies recognize this health disparity, even more so in Canadian [56,65] and Australian [22,66] Indigenous populations.

In Manitoba—a province with one of the highest concentrations of Aboriginal people in Canada—61.2% of Aboriginal women (First Nations, Métis, and Inuit) reported smoking during pregnancy, compared to 26.2% of non-Aboriginal women [56]. This coincides with maternal smoking rates in Australia where one in two (49.3%) Aboriginal and Torres Strait Islander women smoke while pregnant [66] compared to non-Indigenous Australian mothers. 

Such disparities in maternal smoking rates reflect the importance of culturally appropriate cessation resources. Culturally competent risk reduction approaches must therefore be adopted when designing and implementing smoking interventions for these minority populations. To ensure their effectiveness, they must be tailored to the needs of the group they serve, which in this case, means taking into consideration the normality of the use of tobacco, the low SES, and the cultural values and beliefs of Indigenous populations [67].

Although systemic reviews promoting tobacco interventions are readily available, researchers agree that additional interventions are needed to reduce tobacco-related health disparities between Indigenous and non-Indigenous groups [14,68,69], even more so in pregnancy [14]. Unfortunately, interventions designed specifically for pregnant Indigenous women have not been resoundingly successful over the last few years [70,71]. 

Borland *et al*. [72] investigated the adequacy of smoking cessation support available to pregnant and postpartum women in Ontario, Canada and found that Northern and rural communities encountered many barriers impeding service availability and utilization. Firstly, inadequate funding for tobacco control and cessation activities relative to larger urban communities was one of the main shortcomings as told by key informants. Secondly, the geographical dispersion of residents inhabiting these remote locations posed a serious threat to the accessibility to resources. Finally, participants articulated the importance of implementing culturally sensitive practices and tailored interventions. This would mean adopting a perspective that addresses traditional tobacco use, distinguishing between typical cigarette smoking and ceremonial or medicinal tobacco use [73], and adopting a holistic approach to wellbeing inclusive of the family and community [72]. 

In addition to ensuring accessibility and engagement, it is important to have a clear understanding of the determinants of health that lead to the initiation and continuation of tobacco use in Indigenous women of reproductive age. This requires tailoring programs to meet the social and economic pressures experienced by minority groups. Poverty, social stigma and misconceptions regarding the safety of stop-smoking aids are only some of the barriers hindering the success of stop-smoking interventions [72]. Difficult life circumstances and high levels of stress are equally common among Aboriginal pregnant smokers [14] and take precedence over smoking cessation. Furthermore, widespread use of tobacco products among Indigenous people makes it particularly difficult for pregnant women to avoid other smokers and obtain support from family and partners when attempting to quit [74]. 

In their smoking cessation guide, Gould *et al.* [75] suggest that cessation support be available to partners and family members of Aboriginal and Torres Strait Islander women in order to assuage these risks. They also urge that pregnant women be routinely asked about their smoking status, which should be biochemically validated to prevent underreporting. In addition, women should be educated about NRT, along with its risks and benefits, and allowed to make an informed decision. They must be consistently encouraged along the way, no matter how successful they are in their quit attempts and be offered social and community assistance, including Aboriginal specific health and community services that can assist in addressing culturally specific stressors such as financial or housing issues, domestic violence and mental health concerns. As for the health professionals that counsel these women, they require specialized training to teach them about the opportunities and barriers they are likely to encounter along the way. This, along with other evidence-based and culturally competent materials, is how cessation programs tailored for Indigenous women are making strides against maternal smoking [76]. 

### 2.5. Mental Health

A number of studies have compared mental health parameters between pregnant smokers and non-smokers, and found higher rates of depression [77,78,79,80] and a history of mental health problems [25,81] to be important predictors of maternal smoking.

Pregnant smokers report significantly more symptoms of depression and anxiety than spontaneous quitters and are more likely to exhibit social withdrawal [78]. Higher scores on a depression scale are associated with continued smoking during pregnancy, despite controlling for sociodemographic and smoking variables. This suggests that depressive symptoms might be an independent contributor of persistent smoking among expectant mothers. A larger, more recent study of 4295 pregnant women from 15 European countries also reported approximately twice the prevalence of continued smoking among depressed subjects across all European regions [54].

While some studies suggest that a history of depression is associated with a greater difficulty quitting smoking [82], others show no such relationship [25,83]. For example, Gyllstrom and Hellerstedt [24] found that maternal mood did not affect ability to quit, but experiencing stressful life events was a strong determinant. Pregnant women who reported three or more stressful life events were only half as likely to quit smoking as women who reported no stressful events in the previous year. This gives weight to the belief that smoking before pregnancy is a habituated response to the circumstances of women’s lives, such as: unsupportive partners, caring for young children, unstable jobs, domestic situation, and economic vulnerability [84]. Smokers report using tobacco to cope with stress [85,86,87,88], relax, enable time out, and reward themselves [88]. Because short-term nicotine has been shown to alleviate symptoms of ADHD, anxiety, and depression, women may be more likely to transition into chronic tobacco use as a means to self-medicate with nicotine [89]. Patients with mental disorders also report having difficulty quitting smoking because affective symptoms worsen during withdrawal, perpetuating tobacco use.

Overall, there appears to be a lack of robust evidence linking depression and difficulty quitting smoking, warranting the need for further inquiry. Shortcomings in study design have greatly impacted the quality of the available evidence. The majority of literature investigating mental health in pregnant smokers has relied on a cross-sectional design that does not allow for conclusions related to temporality or causality. Methods of data collection are also vulnerable to biases inherent in self-reporting, including recall and social desirability bias. Measuring the level of cotinine, the major metabolite of nicotine, or exhaled carbon monoxide, should have been an integral component in all studies to verify smoking status. Despite being the gold standard, biochemical validation was not included in all studies, which may have been due, in part, to logistical difficulties such as loss of participants to follow-up. In addition, relatively small sample sizes make it difficult to extrapolate larger conclusions regarding the applicability of findings. 

Nonetheless, prenatal depression is an important precursor for postnatal depression [90] and may put the fetus at high risk for prematurity and low birth weight [91,92] in addition to the many adverse health outcomes of smoking during pregnancy. Knowledge about these high-risk groups will have meaningful implications for antenatal care improvement. Even though smoking initiation is multifactorial in origin, several sources point towards negative affect as a vital contributing factor [93]. These individuals might be more susceptible to nicotine dependence, withdrawal, and anxiety symptoms because of its mood altering properties caused by excessive stimulation and reorganization of the brain’s reward circuitry [89,94]. 

It is becoming increasingly clear that smoking and depression have a complex comorbid relationship whereby nicotine intake affects mood, which in turn, increases dependence. If this is indeed the case, then interventions geared towards women of childbearing age should endeavour to reduce depression rates thereby diminishing smoking prevalence among pregnant women [95,96,97]. 

It has been reported that healthcare professionals worry that smoking cessation will worsen ongoing mental illness, which may adversely affect the fetus and that these patients are less motivated to quit. This, in turn, has led to the neglect of smoking cessation for people with mental illness [82,98,99]. 

On the contrary, women who present with symptoms of severe depression may benefit from a depression-focused treatment [95]. Women who tried a depression-focused cessation treatment that specifically targeted interpersonal skills and coping deficits [100], improved in terms of both abstinence and depression. These findings, along with a growing body of evidence, reveal that mood-focused or supportive psychosocial treatments may be helpful for smokers with severe depression, although being of little use to patients with less severe symptoms [101,102,103,104]. Taken together, these demonstrate that incorporating CBT or a more mood-focused approaches as part of smoking cessation strategies, may serve to address the comorbid relationship between persistent tobacco use and mental illness.

### 2.6. Health Services

Pregnancy is regarded as a window of opportunity for quitting smoking, and so, prioritizing successful and enduring tobacco cessation strategies during pregnancy is an ongoing public health concern. Establishing a balanced approach to tobacco cessation strategies that considers social, economic and demographic factors, is informative without being punitive, and produces long-term results with low attrition rates, has proven to be an obstacle for healthcare providers. Health interventions designed over the last three decades have had a poor success rate partly due to the fetus-centric perspective they have adopted [11]. Because approaches to smoking cessation have focused primarily on fetal health, interventions have been confined to the period of pregnancy. For this reason, less attention has been given to the pre- and post- pregnancy time periods, which are equally important opportunities for quitting smoking [58,105]. This may have also served to limit the scope of the interventions, by overlooking important women’s health issues (such as the ones mentioned here) that may have contributed to pregnant women’s inability to quit smoking [11]. In order to ensure treatment adherence, a thorough understanding of the barriers in all areas of antenatal care provision must be addressed: patients, health professionals and health system.

#### 2.6.1. Barriers to Pregnant Smokers

The negative views of women towards healthcare services are an important barrier preventing women from quitting. The judgmental manner in which healthcare professionals behave makes them reluctant to discuss their current smoking behaviour [106]. One in two smokers report that their healthcare providers are either unaware of their smoking or do not discourage smoking during pregnancy [21]. While their motivation to quit is high, their confidence to quit is low [21,107], suggesting the need for support from health professionals. An analysis of the New South Wales Data Collection suggests that delayed attendance for antenatal care is strongly associated with increased risk of smoking in pregnancy [22]. For this reason, investment into improving health service delivery is warranted.

#### 2.6.2. Barriers to Health Professionals and Health System

Current US national guidelines recommend that clinicians provide effective smoking cessation counselling to pregnant women who smoke, utilizing the 5A’s model [108]. This model based on the Agency for Healthcare Research and Quality (AHRQ) guidelines, follows a manualized protocol that includes scripted material and the following steps: Ask about smoking status, Advise to quit smoking, Assess readiness to quit, Assist to quit, and Arrange follow-up. According to a 2013 Cochrane review, behavioural interventions such as the 5A’s increase the smoking abstinence rate by 5% during pregnancy [109]. The 5A’s are currently a best practice approach in the USA for all patients, including pregnant women during prenatal care [110]. Similar approaches have been adopted in Britain [111], Canada [112], and Australia [113].

Unfortunately, a review of healthcare providers’ engagement in smoking cessation with pregnant smokers found that although more than 50% ask women about their smoking status and advise them to quit, fewer than 50% use all components of the 5A’s to address smoking [114]. Factors influencing the provision of smoking cessation counselling based on the 5As model include time constraints [115,116], and inadequate knowledge of cessation interventions and training to implement a brief counselling intervention during pregnancy [116,117]. In line with this, surveys of midwives and gynecologists found that their knowledge of NRT use in pregnancy, was insufficient and thus not recommended to pregnant women [118,119]. “Ask”, “Advise” and to a lesser extent, “Assist” of the 5A’s model, were implemented in smoking cessation communication between these healthcare providers and their clients, but there were important barriers to providing counselling which included lack of time, lack of communication skills in sensitive topics such as smoking cessation and dealing with resistance. When used properly this systematic approach can increase the likelihood that tobacco use is addressed in the healthcare system [108]. Active screening of smoking status among pregnant women during each healthcare visit with accurate methods is crucial to continue and expand efforts. The probability of quitting and readiness to quit increases significantly when asked about smoking by two or more types of health professionals [120], lending support to the routine enquiry about smoking status. Fiore [108] also suggests that such strategies be evaluated and lessons shared, to allow such data to provide optimal patient care and inform policy decisions. 

In addition, health professionals involved in the care of pregnant women must play a more proactive role when consulted on matters of smoking cessation. By providing them with the knowledge and tools to aid in smoking cessation, they can better educate smokers and motivate them to quit. Healthcare workers would therefore benefit from additional University-accredited certificate programs such as the Training Enhancement in Applied Cessation Counselling and Health (TEACH) Project funded by Smoke Free Ontario [121]. A significant number of obstetrician-gynecologists feel the need for additional training on smoking cessation [115], thus, this should be a mandatory part of their training so that they are better equipped when conducting smoking cessation interventions.

Finally, encouraging collaboration between antenatal care providers and non-medical professionals is a valuable step in providing salient cessation support in the outpatient settings of maternity hospitals and other antenatal settings [21].

## 3. Discussion 

In the largest randomized trial of NRT in pregnancy conducted thus far, the rate of smoking cessation was higher at 1 month in the NRT group compared to the placebo group. This was short-lived as only 7.2% of women assigned to nicotine replacement therapy and 2.8% assigned to placebo reported using trial medication for more than 1 month [6]. Although the short-term efficacy of NRT has been documented in clinical trials, long-term abstinence rates are not promising, as most smokers will relapse. We, and others [5,7,8], suggest that treatment failure in pregnancy may stem from a substantial dropout rate. This leads to the inevitable conclusion that before one designs yet another study whereby the researchers simply require a higher dose schedule of NRT, determinants related to the lack of adherence to treatment must first be identified and taken into account in the study design. 

Maternal smoking rates are much higher for women from lower socioeconomic groups as depicted by low-income levels, lower education attainment, and low occupation status. Other determinants of treatment attrition include: nicotine dependence, social support, culture, mental illness, and health services. Pregnant women who are part of socially disadvantaged and economically marginalised groups have disproportionally high smoking rates and yet, they are underreported in cessation research and interventions. To fully capture the effectiveness of interventions initiated during pregnancy and avoid producing biased estimates of risk, future studies must control for a full range of psychosocial factors. 

Existing studies have adopted a narrow view of what is in fact a complex behaviour. The emphasis has concentrated primarily on individualistic behaviour, excluding social determinants entirely. If the majority of pregnant women who quit do so without intervention [122,123,124,125], it is evident that the advice and programming directed at pregnant women should take a different focus. Determinants such as SES, nicotine dependence, social support, culture, mental illness, and health services should be considered when developing and implementing effective promotional strategies to prevent smoking long before pregnancy all the way through to the postnatal period, thus avoiding potential deleterious health outcomes to the fetus altogether [12]. 

This paper summarizes the importance of the determinants of health in developing and implementing successful smoking cessation programs for pregnant women. Due to the non-systematic nature of this review, some empirical evidence may have been overlooked, thus conclusions should be interpreted with caution. Nevertheless, it presents a strong case for future research trials and treatment interventions that will serve to reduce health disparities of maternal smoking.

## 4. Conclusions 

In conclusion, we believe that identifying and addressing the determinants of health associated with tobacco use and relapse rates may ultimately improve efficacy of current pharmacology-driven strategies. Moving forward, it is clear that novel strategies that target the multiple and interacting psychosocial, cultural, economic, and biological barriers to smoking cessation must be implemented in research and treatment development to ensure treatment adherence and long-term cessation. Evidently, strategies may be based on but not limited to those described herein and should be tailored to the needs of the individual seeking treatment. 
